# Prediction of miRNA–Disease Associations by Cascade Forest Model Based on Stacked Autoencoder

**DOI:** 10.3390/molecules28135013

**Published:** 2023-06-27

**Authors:** Xiang Hu, Zhixiang Yin, Zhiliang Zeng, Yu Peng

**Affiliations:** Center of Intelligent Computing and Applied Statistics, School of Mathematics, Physics and Statistics, Shanghai University of Engineering Science, Shanghai 201620, China

**Keywords:** miRNA–disease association, multi-source information, stacked autoencoder, cascade forest

## Abstract

Numerous pieces of evidence have indicated that microRNA (miRNA) plays a crucial role in a series of significant biological processes and is closely related to complex disease. However, the traditional biological experimental methods used to verify disease-related miRNAs are inefficient and expensive. Thus, it is necessary to design some excellent approaches to improve efficiency. In this work, a novel method (CFSAEMDA) is proposed for the prediction of unknown miRNA–disease associations (MDAs). Specifically, we first capture the interactive features of miRNA and disease by integrating multi-source information. Then, the stacked autoencoder is applied for obtaining the underlying feature representation. Finally, the modified cascade forest model is employed to complete the final prediction. The experimental results present that the AUC value obtained by our method is 97.67%. The performance of CFSAEMDA is superior to several of the latest methods. In addition, case studies conducted on lung neoplasms, breast neoplasms and hepatocellular carcinoma further show that the CFSAEMDA method may be regarded as a utility approach to infer unknown disease–miRNA relationships.

## 1. Introduction

MicroRNAs (miRNAs) are a class of short non-coding RNA molecules and play critical roles in gene expression programs and biological processes [1,2,3]. Many scholars have explored and studied the role of miRNAs in the human body [4]. Moreover, some new works in the literature have shown that the differential expression of miRNAs is associated with human disease pathogenesis, such as Alzheimer’s disease [5], cardiovascular disease [6], breast cancer [7,8] and many others [9,10,11]. Therefore, the study of the relationship between miRNA and disease is meaningful for the treatment of diseases and has become a hot topic in the field of bioinformatics.

Some traditional biological experimental methods that are used to verify unknown miRNAs associated with a certain disease, such as reverse transcription polymerase chain reaction [12] and microarray profiling [13], often require a large investment of money and time and are inefficient. Now, the computing efficiency of computers has greatly been improved. We can design some excellent predictive models to identify unknown MDAs, which are efficient and economical. These associations can be used for further experimental verification. The current potential miRNA–disease relationship identification methods can be divided into two types. The first type of method is based on a network algorithm that exploits the similarity of miRNAs and diseases from different perspectives. The pioneering model was designed by Jiang et al. [14]. The model applies a scoring system to rank the probability of disease-related miRNAs. By integrating Gaussian similarity, Chen et al. [15] designed the WBSMDA method to predict MDAs. In addition, due to WBSMDA being a global rank method, this method allows for calculating the connections of miRNA–disease for all diseases simultaneously. Zeng et al. [16] used a bilayer network and applied the structural consistency as a metric of network performance. Then the authors used structural perturbation to infer MDAs in the bilayer network. In [17], via non-negative matrix factorization, the authors proposed a ranking method. In particular, this method introduced the sparseness characteristic to make prediction more reliable. Yu et al. [18] developed KDFGMDA to infer unknown disease-related miRNAs. The KDFGMDA method first obtained triple information of existing databases and massive experimental data, then a graph representation model was trained to complete prediction tasks. Zhang et al. [19] proposed the FLNSNLI method, which is based on fast linear neighborhood similarity. The miRNAs and diseases are expressed in the form association profiles, then the fast linear neighborhood similarity and association profiles are used to measure miRNA–miRNA similarity and disease–disease similarity. Finally, the label propagation algorithm is applied to calculate the probability score. Some other interesting methods based on a complex network can be seen in [20,21].

The second category of methods is machine learning-based methods, which have been indicated to be powerful in many classification tasks, especially in bioinformatics [22,23]. For instance, Zheng et al. [24] developed the MLMDA method to predict microRNA–disease associations. This method used the random forest classifier to predict via integrating heterogeneous information sources. Zhou et al. [25] used K-means clustering to balance the sample set, and used gradient boosting decision trees for feature extraction. Finally, the logical regression is applied to predict labels. Some deep learning methods are also designed for predicting MDAs. Chen et al. [26] inferred novel MDAs by the deep-belief network model. During the pre-training process, this method utilized all of the information. Liu et al. [27] proposed a new feature representation method, and used the random forest method to complete the prediction. Ai et al. [28] designed an end-to-end computational method by integrating the multi-source information and reformulating the score matrix. In [29], based on the deep neural network, the authors developed a new model named NCFM, which integrated the generalization of the neural network and the memorization of matrix factorization. The MDA-CNN method was proposed in [30]. This method first obtains interaction features. Then, an autoencoder is utilized to obtain the critical feature combination. Finally, after inputting the low-dimensional feature representation, MDA-CNN inferred the final label by using a convolutional neural network.Wang et al. [31] fully utilized unlabeled samples and pretrained the stacked autoencoders in an unsupervised manner. This method is suitable for the dataset with a small number of positive data and a large number of negative data.

More and more deep neural networks are used for classification tasks and have achieved good results. Inspired by deep neural networks processing information layer by layer, Zhou et al. [32] developed the deep forest learning model, including the multi-grained scanning module and cascade forest structure. Deep forest methods try to build deep models through non-differentiable modules and have fewer hyperparameters than deep neural networks. Furthermore, it is robust to parameter settings, and thus, even if we do not adjust the parameter settings, it still has excellent performance. Due to this, many various prediction tasks are well solved by using the deep forest method [33]. For instance, Chu et al. [34] developed a cascade forest (DTI-CDF) approach to infer novel interactions between the drug and target. Compared with other methods, such as DDR [35], the DTI-CDF model achieved better predictive performance. Zeng et al. [36] presented the AOPEDF method, an arbitrary-order proximity embedded cascade forest method, for predicting drug–target interactions. The AOPEDF method integrated 15 different types of network information, and a deep cascade forest model with 6 estimators was used to complete the prediction task. In the field of MDAs, Dai et al. [37] proposed the MDA-CF method for predicting unknown MDAs. This method uses multiple types of information to represent the association between miRNA and disease, and applies a cascade forest model with four estimators to infer new MDAs. The prediction efficiency of the MDA-CF method is excellent.

In this work, we present a new method called CFSAEMDA to infer MDAs. In the proposed method, various types of information, including miRNA similarity (functional and Gaussian similarity) and disease similarity (semantic and Gaussian similarity), are integrated to construct a comprehensive feature descriptor of MDAs. Then, the latent feature information is obtained by using the stacked autoencoder. Finally, the modified cascade forest model is trained by inputting reduced features to accurately classify and infer novel MDAs. The average area under the receiver operating characteristic curve (AUC) is 97.67%, and is superior to several of the latest methods and classical machine learning algorithms. In particular, the case studies of lung neoplasms, breast neoplasms and hepatocellular carcinoma show that 49, 50 and 47 of the top 50 predicted relevant miRNAs are confirmed by the latest works in the literature, respectively.

## 2. Results

### 2.1. Evaluation Criteria

In this paper, the sample set is divided into the training set and test set in an 8:2 ratio. The training set is used for training the model, and the test set is used for comparing CFSAEMDA with other methods. To obtain a systematic experimental result, we conduct 4-fold cross validation on the training set to evaluate the performance of CFSAEMDA. The training set is randomly divided into four approximately identical parts. Three parts of them are utilized to train the CFSAEMDA model (training subset), and the residual one is used as the test subset. The final result is obtained by averaging the four test subsets.

Our task is binary classification work. To evaluate the performance of CFSAEMDA fairly, we choose several common metrics, including accuracy (Acc), precision (Pre), sensitivity (Sen), specificity (Spe), the Matthews correlation coefficient (MCC), and the $F_{1}$-score (F1). The equations of these metrics are given by

(1) $$Acc=\frac{TP+TN}{TP+TN+FP+FN},$$

(2) $$Pre=\frac{TP}{TP+FP},$$

(3) $$Sen=\frac{TP}{TP+FN},$$

(4) $$Spe=\frac{TN}{TN+FP},$$

(5) $$MCC=\frac{TP*TN-FP*FN}{\sqrt{\left(TP+FP)(TP+FN)(TN+FP)(TN+FN\right)}},$$

(6) $$F1=\frac{2TP}{2TP+FP+FN},$$

where TP and TN are the number of miRNA–disease with association and miRNA–disease without association cases that were successfully identified, respectively. FP(FN) represents the number of miRNA–disease with (without) association examples that are incorrectly identified.

Furthermore, we also calculate the area under the receiver operating characteristic curve (AUC) and the precision–recall curve (AUPR), respectively. The range of values for AUC and AUPR is [0,1]. Normally, the higher AUPR and AUC values represent the better performance of the model.

### 2.2. Feature Evaluation

**Figure 1:**
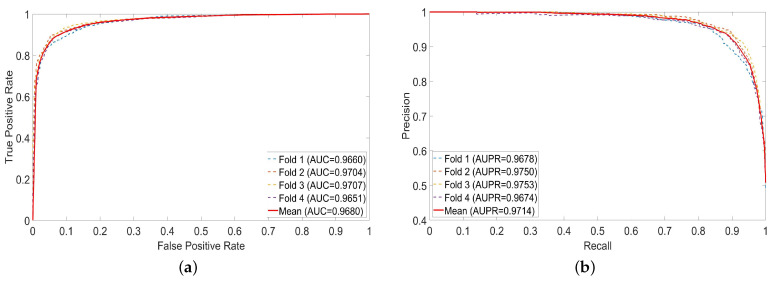
ROC and P-R curves of CFSAEMDA. (**a**) ROC curves; (**b**) P-R curves.

In this section, we present the results of 4-fold cross validation. From Table 1, CFSAEMDA obtains an average AUC of 96.80%, which is the mean of 96.60%, 97.04%, 97.07% and 96.51%, and achieves an AUPR of 97.14%, which is the average of 96.78%, 97.50%, 97.53% and 96.74%. Figure 1 shows the receiver operating characteristic curves and precision–recall curves of the CFSAEMDA method. It can be observed that the ROC curve can reach the upper left corner of the graph, while the P-R curve almost reaches the upper right corner of the graph, indicating the effectiveness of this method. In addition, the results of the average Acc, Pre, Sen, Spe, MCC and F1 are 91.00%, 91.02%, 91.09%, 90.88%, 81.99% and 91.05%, respectively. These results indicate that the CFSAEMDA method has an excellent ability to infer unknown miRNA–disease associations.

### 2.3. Ablation Experiments

In this section, we conduct ablation experiments to verify the effectiveness of our modifications of the estimators and predictor. We design two models: One of them is the traditional cascade forest structure without any modifications (Model 1). The other model only has modification to the estimators (Model 2). The training set is applied to train the model, and the test set is used for model comparison. The experimental results are listed in Table 2.

Regarding the effectiveness of the modification of the estimators, as shown in Table 2, Model 2 can further improve the performance of Acc, Pre, Sen, Spe, MCC, F1, AUPR and AUC. The estimators in Model 2 is more diverse than Model 1, and thus more information can be obtained at each level.

Regarding the effectiveness of the modification of the predictors, as shown in Table 2, CFSAEMDA can further improve the performance of Acc, Pre, Sen, Spe, MCC, F1, AUPR and AUC. Model 1 and Model 2 achieve the final predicted value by averaging the prediction results of the estimators. In the CFSAEMDA method, we modify the predictor to the SVM algorithm, which greatly improves the performance of the model.

### 2.4. Method Comparison

In this section, we compare CFSAEMDA with several of the latest prediction methods, including MDA-CF [37], DDIMDL [38] and AOPEDF [36]. One machine learning method, the SVM algorithm, is also considered. These methods have shown excellent performance in classification tasks, especially in the field of bioinformatics. The MDA-CF method achieved prediction by the cascaded forest model. The estimators in the MDA-CF method are set to two XGBoosts and two random forests. The AOPEDF method also used the cascaded forest model to predict potential MDAs, which includes two random forests, two XGBoosts and two extra trees. The DDIMDL method used a deep neural network to obtain the final results. We modified the code of MDA-CF, DDIMDL, AOPEDF and SVM to adapt to the task proposed in this study and compared them with the proposed CFSAEMDA method. We trained the model under identical conditions and used the test set to evaluate its performance.

**Figure 2:**
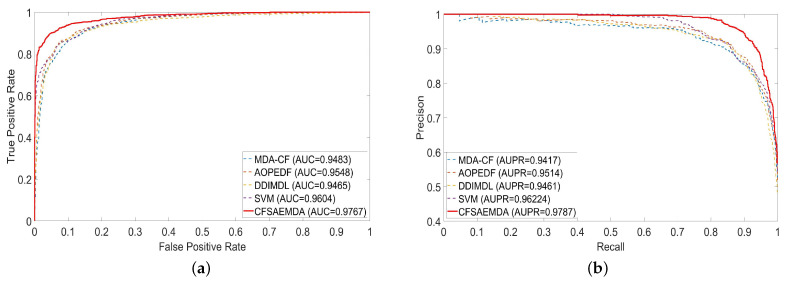
ROC and P-R curves of different methods. (**a**) ROC curves; (**b**) P-R curves.

The predictive performance of each model is shown in Table 3. The CFSAEMDA method obtained an AUC of 97.67%, which is superior to MDA-CF (94.83%), AOPEDF (95.48%), DDIMDL (94.65%) and SVM (96.04%). The value of AUPR of the proposed method is 97.87%, which is superior to MDA-CF (94.17%), AOPEDF (95.14%), DDIMDL (94.61%) and SVM (96.22%). In other metrics, our proposed method also has the best performance. In addition, we also drew the ROC curves and P-R curves of each compared method (Figure 2). It is easy to find that the ROC curve and P-R curve of the proposed method are closer to the upper left and upper right than other methods, which indicates that our method is more efficient.

### 2.5. Case Study

To further indicate the application of CFSAEMDA in practice, case studies on lung neoplasms, breast neoplasms and hepatocellular carcinoma were performed. Many scholars are very interested in the study of diseases in humans, and effective early diagnosis is crucial to the treatment of diseases. We first removed all the association information of specific diseases, including known and unknown associations. Then we balanced the remaining samples for training. Finally, the trained model was used to output the prediction probability scores and sort them. The prediction results were confirmed using the HMDD v3.0 [39] database.

Firstly, we chose to study the associations between lung neoplasms and miRNAs. Lung neoplasms are abnormal growths of tissue that form in the lungs. Among all cancers, the death rate of lung neoplasms is the highest in men and the second highest in women. Numerous works in the literature have shown that the generation of lung neoplasms is associated with some miRNAs. For instance, let-7 is involved in the pathogenesis, migration and diffusion of lung neoplasms [40]. Thus, it is necessary to conduct the investigation on the lung neoplasms-related miRNAs. According to the experimental results, 49 of the top 50 lung neoplasms-associated miRNAs are verified (see Supplementary Table S1).

We then completed the association study of breast neoplasms. Breast neoplasm is cancer of the breast tissues, most commonly arising from the milk ducts. For women, breast neoplasm is the common type of cancer. Therefore, the early diagnosis of breast neoplasm is crucial to the treatment of the disease. The researchers found that breast neoplasm is related to the overexpression of circulating miRNA-146a [41]. Based on the experimental results, 50 of the top 50 breast neoplasm-associated miRNAs are confirmed by database (see Supplementary Table S2).

Finally, hepatocellular carcinoma is chosen as the third case study. hepatocellular carcinoma tends to be the main cause of death for patients with cirrhosis. Among all cancer diseases, hepatocellular carcinoma is the third-leading cause of death. Furthermore, the differential expression of miRNAs contributes to the development of hepatocellular carcinoma. Therefore, we chose hepatocellular carcinoma as a case study. The experimental results show that 47 of the top 50 hepatocellular carcinoma-associated miRNAs are confirmed (see Supplementary Table S3).

It should be pointed out that some novel MDAs were found by our proposed method. For example, hsa-mir-92b was not verified to be associated with breast neoplasms in HMDD v2.0. However, the CFSAEMDA method found this MDA, and HMDD v3.0 confirmed it. Other novel MDAs found by our method are highlighted in Supplementary Tables S1–S3. The case studies show the potential of the CFSAEMDA approach in guiding biological experiments.

## 3. Materials and Methods

### 3.1. Dataset

The dataset used in this paper is obtained from the HMDD v2.0 database [42], comprising 495 miRNAs, 383 diseases and 5430 MDAs that are experimentally verified, and the number of unknown MDAs is 184,155. The adjacency matrix A(i,j) is utilized to represent the interaction information between miRNAs and diseases. If miRNA $m_{i}$ is correlated with disease $d_{j}$, the value of $A_{\left(i,j\right)}$ is equal to 1; otherwise, it is equal to 0. Here, we treat 5430 verified associations as the positive sample set. In order to better reflect the performance of the model, we randomly select 5430 associations from the unknown associations as a negative sample set to balance the sample set (see Table 4).

### 3.2. Multi-Source Information

#### 3.2.1. Functional Similarity of miRNA

Based on the assumption that different miRNAs associated with similar diseases have similar functions, Wang et al. collected and organized the miRNA functional similarity data [43]. The corresponding data can be downloaded from http://www.cuilab.cn/files/images/cuilab/misim.zip, accessed on 28 May 2023. Thus, in this paper, the matrix $MFS(m_{i},m_{j})$ is utilized to describe the functional similarity two miRNAs.

#### 3.2.2. Semantic Similarity of Disease

We apply several directed acyclic graphs (DAGs) to estimate the semantic similarity of diseases [44]. Specifically, each disease *i* can be represented as follows: $DAG_{i}=\left(i,G\left(i\right),E\left(i\right)\right)$. Here, G(i) denotes the node set of all diseases, and E(i) stands for the edges in DAG. Therefore, the semantic contribution value C1 of disease *d* to ancestor node *k* can be computed by

(7) $$C1_{d}\left(k\right)=\left\{\begin{array}{cc} \max\{\mu *C1_{d}\left(k^{\prime }\right)|k^{\prime }\in \mathrm{children}\;\mathrm{of}\;k\},\;\mathrm{if}\;d\;\neq \;k & \;\\ 1,\;\mathrm{if}\;d\;=\;k \end{array}\right.,$$

where μ denotes the semantic contribution factor and is equal to 0.5 [43]. Then the disease semantic value DS1(d) is given by

(8) $$DS1\left(d\right)=\sum_{k\in G(d)}C1_{d}\left(k\right).$$

The disease semantic similarity DSS1 is described by

(9) $$DSS1\left(d_{i},d_{j}\right)=\frac{\sum_{k\in G\left(d_{i}\right)\cap G\left(d_{j}\right)}\left(C1_{d_{i}}\left(k\right)+C1_{d_{j}}\left(k\right)\right)}{DS1\left(d_{i}\right)+DS1\left(d_{j}\right)},$$

where $d_{i}$ and $d_{j}$ represent two diseases.

However, the diseases’ semantic contribution value is different at the same level in the DAGs. Thus another method is applied to calculate the semantic similarity of diseases [45]; the specific formula is given by

(10) $$C2_{d}\left(k\right)=-\log\frac{NG(k)}{nd},$$

where nd is the number of diseases, and NG(k) represents the number of DAGs, including *k*.

Similarly, the disease semantic value DS2(d) and the disease semantic similarity DSS2 can be calculated by

(11) $$DS2\left(d\right)=\sum_{k\in G(d)}C2_{d}\left(k\right),$$

(12) $$DSS2\left(d_{i},d_{j}\right)=\frac{\sum_{k\in G\left(d_{i}\right)\cap G\left(d_{j}\right)}\left(C2_{d_{i}}\left(k\right)+C2_{d_{j}}\left(k\right)\right)}{DS2\left(d_{i}\right)+DS2\left(d_{j}\right)}.$$

Therefore, the final disease semantic similarity DSS is given by

(13) $$DSS\left(d_{i},d_{j}\right)=\frac{1}{2}\left(DSS1\left(d_{i},d_{j}\right)+DSS2\left(d_{i},d_{j}\right)\right).$$

#### 3.2.3. Gaussian Interaction Profile Kernel Similarity of miRNAs and Diseases

According to the literature [46], it can be found that functionally similar miRNAs are more likely to be associated with phenotypically similar diseases. The Gaussian interaction profile (GIP) kernel similarity between miRNAs $m_{i}$ and $m_{j}$ can be established by the following equation:

(14) $$KSM\left(m_{i},m_{j}\right)=\exp(-\alpha_{m}||IP\left(m_{i}\right)-IP\left(m_{j}\right){\left|\right|}^{2}),$$

(15) $$\alpha_{m}=\frac{\alpha_{m}^{\prime }}{\frac{1}{nm}\sum_{i=1}^{nm}||IP\left(m_{i}\right){\left|\right|}^{2}},$$

where $\alpha_{m}$ controls the kernel bandwidth, $IP(m_{i})$ and $IP(m_{j})$ denote the *i*th and *j*th rows in matrix IP, nm is the number of miRNAs, and $\alpha_{m}^{\prime }$ is equal to 1 [47].

Similarly, the GIP kernel similarity between diseases $d_{i}$ and $d_{j}$ is calculated as follows:

(16) $$KSD\left(d_{i},d_{j}\right)=\exp(-\alpha_{d}||IP\left(d_{i}\right)-IP\left(d_{j}\right){\left|\right|}^{2}),$$

(17) $$\alpha_{d}=\frac{\alpha_{d}^{\prime }}{\frac{1}{nd}\sum_{i=1}^{nd}||IP\left(d_{i}\right){\left|\right|}^{2}}.$$

### 3.3. Integrated Similarity Characteristic

For miRNAs, we collected the functional similarity information and the GIP kernel similarity information. Considering the lack of functional similarity of some miRNAs, the miRNA similarity is calculated by

(18) $$MS\left(m_{i},m_{j}\right)=\left\{\begin{array}{c} \frac{KSM\left(m_{i},m_{j}\right)+MFS\left(m_{i},m_{j}\right)}{2},\;\mathrm{if}\;MFS\left(m_{i},m_{j}\right)\;\mathrm{exists}\;\\ KSM(m_{i},m_{j}),\;\mathrm{otherwise} \end{array}\right..$$

Similarly, the following formula is applied to compute the disease similarity:

(19) $$DS\left(d_{i},d_{j}\right)=\left\{\begin{array}{c} \frac{KSD\left(d_{i},d_{j}\right)+DSS\left(d_{i},d_{j}\right)}{2},\;\mathrm{if}\;DSS\left(d_{i},d_{j}\right)\;\mathrm{exists}\;\\ KSD(d_{i},d_{j}),\;\mathrm{otherwise} \end{array}\right..$$

### 3.4. CFSAEMDA

**Figure 3:**
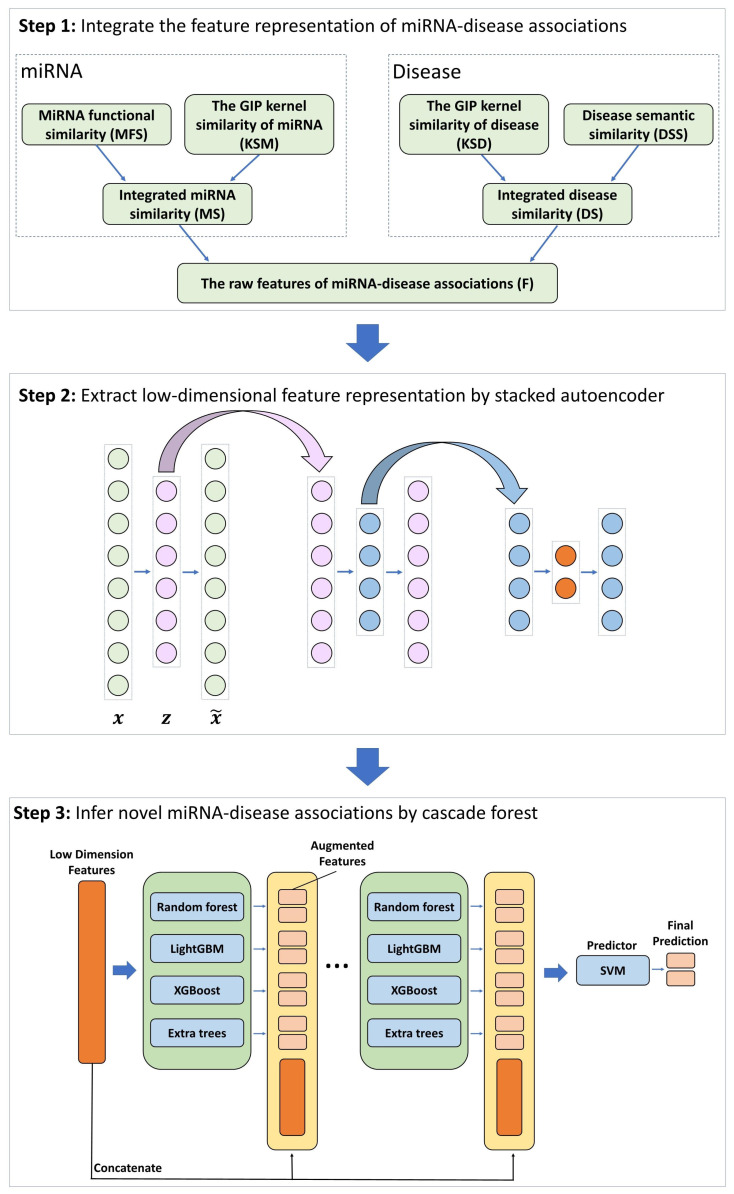
The flowchart of CFSAEMDA.

The present paper introduces a new approach called CFSAEMDA for predicting disease-related miRNAs, which contains three main steps: (i) this paper first construct a comprehensive feature representation of miRNA and disease based on multi-source information. (ii) the stacked autoencoder is applied to extract latent information representation, and (iii) the potential MDAs are predicted by a modified cascade forest structure. The framework of CFSAEMDA is shown in Figure 3.

#### 3.4.1. Feature Representation

In this part, inspired by [27], the following strategy is used to construct the high-quality feature representation of MDAs. We obtained the 495-dimensional vector $MS_{ij}$ representing the miRNA similarity network information between two miRNAs. The 383-dimensional vector $DS_{ij}$ for each disease is also obtained. Then, the feature representation of miRNAs ($F_{m}$) and disease ($F_{d}$) are given by

(20) $$F_{m}={\left(MS_{1}D_{1}^{\prime },\cdots ,MS_{1}D_{383}^{\prime },\cdots ,MS_{495}D_{1}^{\prime },\cdots ,MS_{495}D_{383}^{\prime }\right)}^{T},$$

(21) $$F_{d}={\left(DS_{1}D_{1},\cdots ,DS_{1}D_{495},\cdots ,DS_{383}D_{1},\cdots ,DS_{383}D_{495}\right)}^{T},$$

where the matrix *D* is the verified MDA network, $D_{i}$ is the *i*th row of *D* and $D_{j}^{\prime }$ is the *j*th column transpose of *D*. $F_{m}$ is the feature representation of miRNAs with 495 × 383 row and 495 + 495 column. $F_{d}$ is the feature representation of disease with 495 × 383 row and 383 + 383 column. The final high-quality feature representation of MDAs is given by the following formula:

(22) $$F=(F_{m},F_{d}),$$

where *F* is a matrix with 189,585 (495 × 383) rows and 1756 (495 + 495 + 383 + 383) columns. Then, we randomly select 5430 associations from the unknown associations as a negative sample set to balance the sample set. Therefore, we have 10,860 samples with 1756 dimensional features.

#### 3.4.2. Stacked Autoencoder

Machine learning models have high requirements for the dimensionality of the data input. Namely, the high-dimensional features seriously influence the prediction performance. The feature representation obtained in the previous section is of 1756 dimensions, which is highly dimensional. Therefore, in this article, the stacked autoencoder is utilized to learn the latent feature vectors.

The stacked autoencoder is a deep learning model constructed by stacking multiple single autoencoders [48]. An autoencoder consists of an encoder and a decoder. In the encoding phase, the original feature representation is fed to the encoder to achieve feature compression and dimensionality reduction. The corresponding formula is given by

(23) $$z=f_{e}\left(wx+b\right),$$

where *x* is the original high-dimensional feature input, *w* and *b* denote the weights and bias, $f_{e}\left(•\right)$ represents the non-linear activation function of the encoder phase, and *z* is the output of the encoder.

The purpose of the decoder is to use the latent representation *z* to reconstruct the input *x*. The corresponding formula is given by

(24) $$\tilde{x}=f_{d}\left(w^{\prime }z+b^{\prime }\right),$$

where $\tilde{x}$ is the reconstruction feature representation of input *x*; $w^{\prime }$ and $b^{\prime }$ represent the weight and bias of the decoder phase; and $f_{d}\left(•\right)$ denotes the non-linear activation function.

Finally, the autoencoder is trained to minimize the reconstruction errors, and the formula is given by

(25) $$l\left(x,\tilde{x}\right)=\sum_{i=1}^{N}\left|\right|x_{i}-\tilde{x_{i}}{\left|\right|}^{2},$$

where *N* is the number of sample sets. The autoencoder is trained to minimize the above loss function and update all parameters iteratively.

Here, according to previous literature [49], the stacked autoencoder is constructed by stacking three of the same autoencoders. The latent feature output dimensions of the three autoencoders are set to 1024, 512 and 256, respectively. Namely, the dimension of reduced feature representations is 256. In addition, the Adam optimizer [50], an algorithm for first-order gradient-based optimization of stochastic objective functions based on low order moment adaptive estimation, is used to update the parameters in the training process of the stacked autoencoder. The Adam optimizer is better than the stochastic gradient descent optimizer. In each hidden layer, the activation function is set to the tanh activation function, and the formula is given by

(26) $$\tanh\left(x\right)=\frac{e^{x}-e^{-x}}{e^{x}+e^{-x}}.$$

#### 3.4.3. Modified Cascade Forest Structure

In this article, after extracting the latent vector representation, the cascade forest structure (see Figure 3) is utilized for prediction. This structure includes two parts: estimators and predictor, which can ensure the layer-by-layer processing of information.

**Figure 4:**
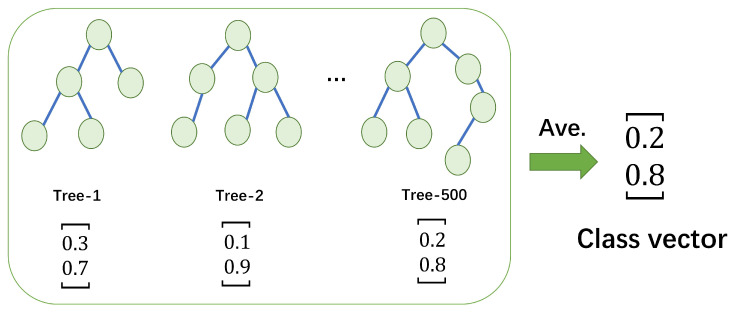
Illustration of class vector generation.

Here, at each level of cascade, we set four estimators, including one random forest [51], one completely random tree forest, one XGBoost [52] and one LightGBM [53]. Each estimator has 100 trees, and there are 400 trees in each cascaded level. The diversity of estimators is crucial for ensemble construction [54]. Given an instance, each estimater can produce an estimate of class distribution. For example, the random forest estimator averages the results of each tree to obtain the final class vector as illustrated in Figure 4. In our work, it is a binary classification task. Four estimators will generate eight-dimensional class vectors, which can be regarded as augmented features. Then, the class vectors are concatenated with the original feature vectors. In addition, each estimator will conduct 5-fold cross validation to prevent overfitting. After expanding to a new level, the performance of the entire cascade will be evaluated on the validation set. If there is no significant improvement in the performance, the cascade layer will automatically terminate. Namely, the complexity of the cascade forest model is determined by the datasets.

The traditional cascaded forest model obtains the final predicted value by averaging the prediction results of four estimators. In this paper, we modify the predictor to the support vector machine (SVM) algorithm [55], which has excellent predictive ability. The kernel function of SVM is set to the polynomial function. The modification of the predictor greatly improves the performance of the model.

## 4. Discussion and Conclusions

In this work, we propose a novel computational model, called CFSAEMDA, to infer unknown MDAs. First, the association between diseases and miRNAs can be represented by integrating multi-source similarity features, including functional and GIP kernel similarity of miRNA, semantic and GIP kernel similarity of disease. Then the stacked autoencoder is applied to extract the latent representation of feature. Finally, a modified cascade forest model is employed for predicting the final labels of MDAs. The experimental results demonstrate that our model is excellent and stable. Three case studies further show that CFSAEMDA can be utilized to guide biological experiments. In conclusion, CFSAEMDA is a valuable and convenient method to infer novel MDAs.

## Figures and Tables

**Table 1:** Performance of CFSAEMDA.

Fold	Acc (%)	Pre (%)	Sen (%)	Spe (%)	MCC (%)	F1 (%)	AUPR (%)	AUC (%)
1	89.73	89.84	89.08	90.36	79.45	89.46	96.78	96.60
2	91.48	91.86	91.45	91.52	82.96	91.66	97.50	97.04
3	91.94	91.40	93.12	90.69	83.88	92.25	97.53	97.07
4	90.84	90.98	90.73	90.95	81.68	90.85	96.74	96.51
Mean	91.00	91.02	91.09	90.88	81.99	91.05	97.14	96.80

**Table 2:** Performance of ablation experiments.

Method	Acc (%)	Pre (%)	Sen (%)	Spe (%)	MCC (%)	F1 (%)	AUPR (%)	AUC (%)
Model 1	87.52	87.91	85.99	88.96	75.02	86.94	93.81	94.57
Model 2	88.63	89.78	86.27	90.83	77.26	87.99	94.91	95.35
CFSAEMDA	92.27	92.56	91.33	93.14	84.51	91.94	97.87	97.67

**Table 3:** The results of different methods.

Method	Acc (%)	Pre (%)	Sen (%)	Spe (%)	MCC (%)	F1 (%)	AUPR (%)	AUC (%)
MDA-CF	87.75	86.15	88.94	86.64	75.54	87.52	94.17	94.83
AOPEDF	88.86	89.75	86.84	90.74	77.70	88.28	95.14	95.48
DDIMDL	88.35	86.92	89.32	87.44	76.73	88.11	94.61	94.65
SVM	88.21	88.16	87.32	89.05	76.40	87.74	96.22	96.04
CFSAEMDA	92.27	92.56	91.33	93.14	84.51	91.94	97.87	97.67

**Table 4:** Summary of the samples on the original and balanced datasets.

Data	Known Associations	Unknown Associations
Original dataset	5430	184,155
Balanced Dataset	5430	5430

## Data Availability

We provided the python data and code for CFSAEMDA at https://github.com/huxiang666666/CFSAEMDA, accessed on 28 May 2023.

## Supplementary Materials

The following supporting information can be downloaded at: https://www.mdpi.com/article/10.3390/molecules28135013/s1, Table S1: The prediction results of lung neoplasms; Table S2: The prediction results of breast neoplasms; Table S3: The prediction results of hepatocellular carcinoma.
