# Supplementary material for: Prediction of miRNA–Disease Associations by Cascade Forest Model Based on Stacked Autoencoder
Source: Molecules. 2023 Jun 27;28(13):5013. doi: 10.3390/molecules28135013 (PMC10343850; doi:10.3390/molecules28135013)
Supplement: Supplementary file 1 [file molecules-28-05013-s001.zip › Supplementary Table S1.pdf]

The prediction results of lung neoplasms

1	hsa-mir-155	Confirmed
2	hsa-mir-19a	Confirmed
3	hsa-mir-20a	Confirmed
4	hsa-mir-16	Unconfirmed
5	hsa-mir-21	Confirmed
6	hsa-mir-223	Confirmed
7	hsa-mir-17	Confirmed
8	hsa-mir-18a	Confirmed
9	hsa-mir-146a	Confirmed
10	hsa-mir-92a	Confirmed
11	hsa-mir-150	Confirmed
12	hsa-mir-1	Confirmed
13	hsa-let-7a	Confirmed
14	hsa-mir-183	Confirmed
15	hsa-mir-24	Confirmed
16	hsa-mir-200b	Confirmed
17	hsa-let-7b	Confirmed
18	hsa-mir-133a	Confirmed
19	hsa-mir-29a	Confirmed
20	hsa-mir-148a	Confirmed
21	hsa-let-7d	Confirmed
22	hsa-let-7g	Confirmed
23	hsa-mir-192	Confirmed
24	hsa-mir-200c	Confirmed
25	hsa-mir-181b	Confirmed
26	hsa-mir-26a	Confirmed
27	hsa-mir-133b	Confirmed
28	hsa-mir-34a	Confirmed
29	hsa-mir-15b	Confirmed
30	hsa-mir-125b	Confirmed
31	hsa-mir-15a	Confirmed
32	hsa-mir-196a	Confirmed
33	hsa-mir-200a	Confirmed
34	hsa-mir-19b	Confirmed
35	hsa-let-7e	Confirmed
36	hsa-mir-143	Confirmed
37	hsa-let-7c	Confirmed
38	hsa-mir-126	Confirmed
39	hsa-mir-106a	Confirmed
40	hsa-mir-195	Confirmed
41	hsa-let-7f	Confirmed
42	hsa-mir-122	Confirmed
43	hsa-mir-142	Confirmed

44	hsa-mir-29b	Confirmed
45	hsa-mir-30b	Confirmed
46	hsa-mir-101	Confirmed
47	hsa-mir-203	Confirmed
48	hsa-mir-221	Confirmed
49	hsa-mir-205	Confirmed
50	hsa-mir-182	Confirmed
