# Supplementary material for: Prediction of miRNA–Disease Associations by Cascade Forest Model Based on Stacked Autoencoder
Source: Molecules. 2023 Jun 27;28(13):5013. doi: 10.3390/molecules28135013 (PMC10343850; doi:10.3390/molecules28135013)
Supplement: Supplementary file 1 [file molecules-28-05013-s001.zip › Supplementary Table S2.pdf]

The prediction results of breast neoplasms

1	hsa-mir-21	Confirmed
2	hsa-mir-155	Confirmed
3	hsa-mir-16	Confirmed
4	hsa-mir-221	Confirmed
5	hsa-mir-200b	Confirmed
6	hsa-let-7a	Confirmed
7	hsa-mir-145	Confirmed
8	hsa-mir-9	Confirmed
9	hsa-mir-1	Confirmed
10	hsa-mir-133a	Confirmed
11	hsa-mir-19a	Confirmed
12	hsa-mir-200a	Confirmed
13	hsa-mir-133b	Confirmed
14	hsa-mir-19b	Confirmed
15	hsa-mir-146a	Confirmed
16	hsa-mir-218	Confirmed
17	hsa-mir-222	Confirmed
18	hsa-mir-92b	Confirmed
19	hsa-let-7c	Confirmed
20	hsa-mir-141	Confirmed
21	hsa-mir-126	Confirmed
22	hsa-mir-125b	Confirmed
23	hsa-mir-199a	Confirmed
24	hsa-mir-200c	Confirmed
25	hsa-mir-302b	Confirmed
26	hsa-mir-214	Confirmed
27	hsa-mir-205	Confirmed
28	hsa-mir-22	Confirmed
29	hsa-mir-96	Confirmed
30	hsa-let-7b	Confirmed
31	hsa-mir-17	Confirmed
32	hsa-mir-375	Confirmed
33	hsa-mir-29b	Confirmed
34	hsa-mir-302c	Confirmed
35	hsa-mir-181a	Confirmed
36	hsa-let-7f	Confirmed
37	hsa-mir-146b	Confirmed
38	hsa-mir-31	Confirmed
39	hsa-mir-106b	Confirmed
40	hsa-mir-302d	Confirmed
41	hsa-mir-125a	Confirmed
42	hsa-mir-210	Confirmed
43	hsa-mir-34b	Confirmed

44	hsa-mir-302a	Confirmed
45	hsa-mir-34a	Confirmed
46	hsa-mir-193b	Confirmed
47	hsa-let-7i	Confirmed
48	hsa-mir-150	Confirmed
49	hsa-mir-199b	Confirmed
50	hsa-mir-486	Confirmed
