# Supplementary material for: Prediction of miRNA–Disease Associations by Cascade Forest Model Based on Stacked Autoencoder
Source: Molecules. 2023 Jun 27;28(13):5013. doi: 10.3390/molecules28135013 (PMC10343850; doi:10.3390/molecules28135013)
Supplement: Supplementary file 1 [file molecules-28-05013-s001.zip › Supplementary Table S3.pdf]

The prediction results of hepatocellular carcinoma

1	hsa-mir-15b	Confirmed
2	hsa-mir-214	Confirmed
3	hsa-mir-182	Confirmed
4	hsa-mir-100	Confirmed
5	hsa-mir-16	Confirmed
6	hsa-mir-26a	Confirmed
7	hsa-mir-183	Confirmed
8	hsa-mir-124	Confirmed
9	hsa-mir-23b	Confirmed
10	hsa-mir-181a	Confirmed
11	hsa-let-7d	Confirmed
12	hsa-mir-101	Confirmed
13	hsa-mir-34c	Confirmed
14	hsa-mir-223	Confirmed
15	hsa-mir-133a	Unconfirmed
16	hsa-mir-486	Confirmed
17	hsa-mir-9	Unconfirmed
18	hsa-mir-497	Confirmed
19	hsa-mir-103a	Confirmed
20	hsa-mir-199a	Confirmed
21	hsa-mir-499a	Confirmed
22	hsa-mir-375	Confirmed
23	hsa-mir-23a	Confirmed
24	hsa-let-7e	Confirmed
25	hsa-mir-137	Confirmed
26	hsa-mir-193a	Confirmed
27	hsa-mir-146a	Confirmed
28	hsa-mir-27a	Confirmed
29	hsa-mir-106a	Confirmed
30	hsa-mir-24	Confirmed
31	hsa-mir-34b	Confirmed
32	hsa-mir-30e	Confirmed
33	hsa-mir-20b	Confirmed
34	hsa-mir-96	Confirmed
35	hsa-mir-133b	Confirmed
36	hsa-mir-22	Confirmed
37	hsa-let-7i	Confirmed
38	hsa-mir-144	Confirmed
39	hsa-let-7c	Confirmed
40	hsa-mir-107	Confirmed
41	hsa-mir-138	Confirmed
42	hsa-mir-424	Confirmed
43	hsa-mir-122	Confirmed

44	hsa-mir-200c	Confirmed
45	hsa-mir-150	Confirmed
46	hsa-mir-449b	Confirmed
47	hsa-mir-302a	Unconfirmed
48	hsa-let-7b	Confirmed
49	hsa-mir-1	Confirmed
50	hsa-mir-151a	Confirmed
